# A simple, field-applicable method to increase the infectivity of wild isolates of *Plasmodium falciparum* to mosquito vectors

**DOI:** 10.1186/s12936-024-04969-0

**Published:** 2024-05-06

**Authors:** Seydou Bienvenu Ouattara, Domonbabele F. D. S. Hien, Ekôbié T. Nao, Prisca S. L. Paré, Edwige Guissou, Anna Cohuet, Isabelle Morlais, Rakiswendé S. Yerbanga, Kounbobr R. Dabiré, Jean Bosco Ouédraogo, Karine Mouline, Thierry Lefèvre

**Affiliations:** 1 Institut de Recherche en Sciences de La Santé, Direction Régionale de L’Ouest (IRSS-DRO), Bobo-Dioulasso, Burkina Faso; 2 Institut Des Sciences Et Techniques (INSTech Bobo), Bobo-Dioulasso, Burkina Faso; 3 Centre Hospitalier Régional de Gaoua (CHRG), Gaoua, Burkina Faso; 4 MIVEGEC, University of Montpellier, IRD, CNRS, Montpellier, France; 5 Université Nazi Boni (UNB), Bobo-Dioulasso, Burkina Faso; 6 Ecole Normale Supérieure, BP 376, Koudougou, Burkina Faso

**Keywords:** *Plasmodium falciparum*, *Anopheles gambiae*, DMFA, Experimental infections, Transmission-blocking interventions, Gametocyte infectivity

## Abstract

**Background:**

The direct membrane feeding assay (DMFA), whereby gametocyte-infected blood is collected from human donors and from which mosquitoes feed through a membrane, is proving essential for assessing parameters influencing *Plasmodium* transmission potential in endemic countries. The success of DMFAs is closely tied to gametocyte density in the blood, with relatively high gametocytaemia ensuring optimal infection levels in mosquitoes. As transmission intensity declines with control efforts, the occurrence of asymptomatic individuals with low gametocyte densities, who can significantly contribute to the infectious reservoir, is increasing. This poses a limitation to studies relying on the experimental infection of large numbers of mosquitoes with natural isolates of *Plasmodium*. A simple, field-applicable method is presented for improving parasite infectivity by concentrating *Plasmodium falciparum* gametocytes.

**Methods:**

*Anopheles gambiae* received one of the following 5 blood treatments through DMFA: (i) whole blood (WB) samples from naturally-infected donors; (ii) donor blood whose plasma was replaced with the same volume of *Plasmodium*-naive AB + serum (1:1 control); (iii) plasma replaced with a volume of malaria-naïve AB + serum equivalent to half (1:1/2), or to a quarter (1:1/4), of the initial plasma volume; and (v) donor blood whose plasma was fully removed (RBC). The experiment was repeated 4 times using 4 distinct wild parasite isolates. Seven days post-infection, a total of 1,095 midguts were examined for oocyst presence.

**Results:**

Substituting plasma with reduced amounts (1:1/2 and 1:1/4) of *Plasmodium*-naive AB + serum led to a 31% and 17% increase of the mosquito infection rate and to a 85% and 308% increase in infection intensity compared to the 1:1 control, respectively. The full removal of plasma (RBC) reduced the infection rate by 58% and the intensity by 64% compared to the 1:1 control. Reducing serum volumes (1:1/2; 1:1/4 and RBC) had no impact on mosquito feeding rate and survival when compared to the 1:1 control.

**Conclusions:**

Concentrating gametocytic blood by replacing natural plasma by lower amount of naive serum can enhance the success of mosquito infection. In an area with low gametocyte density, this simple and practical method of parasite concentration can facilitate studies on human-to-mosquito transmission such as the evaluation of transmission-blocking interventions.

**Supplementary Information:**

The online version contains supplementary material available at 10.1186/s12936-024-04969-0.

## Background

Conducting experimental infections of *Anopheles* mosquito vectors with *Plasmodium* parasites is pivotal in advancing both fundamental and applied malariology. Controlled laboratory infections are essential for unraveling the intricacies of the ecology and evolution of *Anopheles*-*Plasmodium* interactions. In particular, experimental infections have proven instrumental in characterizing the sporogonic cycle and gaining deeper insights into the biology of each developmental stage within the mosquito vector. This has also allowed researchers to accurately evaluate the vector competence of diverse *Anopheles* species and populations, investigate mosquito immune responses and mechanisms of parasite evasion, and evaluate the efficacy of transmission-blocking drugs and vaccines. Finally, experimental infections are indispensable for assessing the impact of *Plasmodium* on mosquito fitness and behaviour (*i.e.* parasite virulence).

Gametocytes, which develop from a subset of asexual parasites in the human bloodstream, are the sexual stages of the parasite responsible for transmission to mosquito vectors. Once ingested by mosquitoes, gametocytes undergo activation within the mosquito gut, and micro- (male) and macro- (female) gametes egress from the enveloping erythrocytes and fertilize in the mosquito midgut. Within a few hours, the resulting zygotes complete a meiosis and develop into motile ookinetes that cross the midgut wall of the mosquito vector and lodge to the basal lamina where they mature into oocysts. Within the oocysts, mitotic divisions produce large number of sporozoites, which break out into the mosquito body cavity, and invade the mosquito salivary glands, rendering the mosquito capable of transmitting the parasites to humans.

Standard Membrane Feeding Assays (SMFA), involving the direct feeding of mosquitoes with culture of *Plasmodium falciparum* gametocytes stands as the preeminent standard for experimental infections worldwide [1–3]. However, SMFA can only use a limited range of cultivable parasite strains and mosquito colonies and, therefore, does not account for genetic diversity, vector-parasite co-evolution, and environmental factors. In contrast, Direct Feeding Assays (DFA) allow mosquitoes to feed directly on the skin of patients carrying gametocytes, the transmissible stage of the parasite [4–6]. Although this approach closely mimics the natural mosquito infection route, DFA poses significant ethical challenges [7, 8]. Direct Membrane Feeding Assays (DMFA) present a compelling compromise between SMFA and DFA [9–12]. DMFA employs gametocyte-infected blood drawn from naturally infected patients in endemic regions, allowing mosquitoes to feed through a membrane. This method embraces human, mosquito, and parasite diversity by conducting assays with blood from various gametocyte-infected volunteers and using sympatric mosquito populations, thereby enabling testing the extrapolation of results obtained with SMFA to the natural transmission system. Importantly, unlike DFA, DMFA allows for the testing of multiple experimental conditions on a single blood sample, thus minimizing uncontrolled variations.

The success of the experimental infection is evaluated by gauging the infection rate and intensity within the mosquito population, through the detection of oocysts or sporozoites approximately 6–8 days or 12–16 days post-infectious blood meal, respectively [3, 9, 11, 12]. The level of infection within the mosquito population in terms of both prevalence and intensity is primarily linked to the density of mature gametocytes circulating in the blood of the human host [13–15]. However, not all gametocyte carriers are necessarily infectious to mosquitoes, as several other factors—whether they stem from the parasite itself (*e.g*., gametocyte sex ratio and maturity), the human host (*e.g.* genetic background, natural transmission-blocking immunity), or the mosquito host (inter and intra-specific genetic variation, immunity)—can influence the establishment of an infection and the ensuing sporogonic development [16].

Gametocyte carriage generally ranges from 5 to 20% among *P. falciparum*-positive individuals and it varies with numerous factors, including host age, genetics, season, transmission intensity, multiplicity of infection, and co-infections with other pathogens [16]. Typically, areas with low transmission intensity exhibit lower gametocyte carriage compared to high-transmission areas [16]. As malaria control efforts ramp up, these low-transmission settings, associated with relatively lower gametocyte carriage and asymptomatic infections, are expected to become more frequent. The identification and quantification of asymptomatic individuals carrying low gametocyte densities become increasingly crucial, due to their significant contribution to the overall infectious reservoir [17]. Understanding the dynamics of transmission in such settings is essential for guiding targeted intervention strategies aimed at interrupting malaria transmission and achieving sustainable control and elimination goals [17].

The selection of high gametocytaemia, crucial for successful mosquito infections, is more challenging in low-transmission settings and can pose a constraint on studies involving substantial numbers of infected mosquitoes. Accordingly, refinements or technical adaptations of DMFA are needed to increase the level of mosquito infection.

A study by Reuling et al*.* [18] investigated three distinct concentration techniques of *P. falciparum* gametocyte-infected whole blood samples. These methods encompassed (i) standard centrifugation, (ii) Percoll gradient, and (iii) magnetic cell sorting (MACS) enrichment, all aimed at augmenting transmission efficiency in mosquito-feeding assays. Their findings demonstrated that while the Percoll gradient and standard centrifugation approaches yielded variable infectivity levels, the MACS gametocyte enrichment consistently surpassed the control in terms of mean abundance of oocysts. Notably, this investigation employed cultured gametocytes of the NF54 *P. falciparum* strain (SMFA), and the effective concentration method necessitated the utilization of a commercial equipment system (specifically, the QuadroMACS™ separator and LS MACS columns).

This study presents a practical and readily implementable method to amplify parasite infectivity to mosquitoes by concentrating natural *P. falciparum* gametocytes. The approach consists in substituting plasma from gametocyte carriers with lower volumes of serum from malaria-naïve donors. Through this method, the infectivity of wild isolates of *P. falciparum* with initial low gametocyte densities to *Anopheles gambiae* in Burkina Faso was effectively increased.

## Methods

### Mosquitoes

Laboratory-reared *Anopheles gambiae* were obtained from an outbred colony established in 2019 from wild-caught mosquitoes collected in Soumousso (11°23′14″N, 4°24′42″W), 40 km from Bobo Dioulasso, south-western Burkina Faso (West Africa), and identified by routine SINE PCR [19]. Mosquitoes were held in 30 cm × 30 cm × 30 cm mesh-covered cages at the IRSS insectary under standard conditions (27 ± 2 °C, 70 ± 5% RH, 12:12 LD). For colony maintenance, adult mosquitoes were fed with a 5% glucose solution and females were provided rabbit blood by direct feeding every three days for oviposition (protocol approved by the national committee of Burkina Faso; IRB registration #00004738 and FWA 00007038). Larvae were reared at a density of about 300 first-instar larvae in 700 mL of water in plastic trays and fed with Tetramin Baby Fish Food (Tetrawerke, Melle, Germany). Upon emergence, three-to-five-day-old females were transferred to paper cups (80–90 females per cup) and starved for 24 h before being exposed to blood meals containing *P. falciparum* gametocytes.

### Mosquito experimental infection

Female *An. gambiae* were fed with blood drawn from naturally infected *P. falciparum* gametocyte-donors recruited among 5 to 12-year-old school children in villages surrounding Bobo-Dioulasso, Burkina Faso using direct membrane feeding assays (DMFA) as previously described [20, 21]. Parasitological screenings were carried out in collaboration with the medical team in charge of malaria treatment at the local health center in these villages. Briefly, thick blood smears were taken from each volunteer, stained with 10% Giemsa and examined under a microscope for observation of all stages of *P. falciparum*. Considering a standard number of 8000 leukocytes/μL of blood, parasite density was assessed against 200 leukocytes for asexual stages and against 1000 leukocytes for gametocytes. Children with asexual parasitaemia of > 1000 parasites per microlitre were treated in accordance with national guidelines. Asymptomatic *P. falciparum* gametocyte-positive children with an initial gametocytaemia of ≤ 80 gametocytes μL^−1^ of blood were recruited. Blood from gametocyte carriers was collected by venipuncture in heparinized tubes. Four distinct parasite isolates, denoted A to D and with a respective gametocytaemia of 32, 40, 72 and 80 gametocytes μL^−1^ of blood, were used for the experimental infections.

**Fig. 1:**
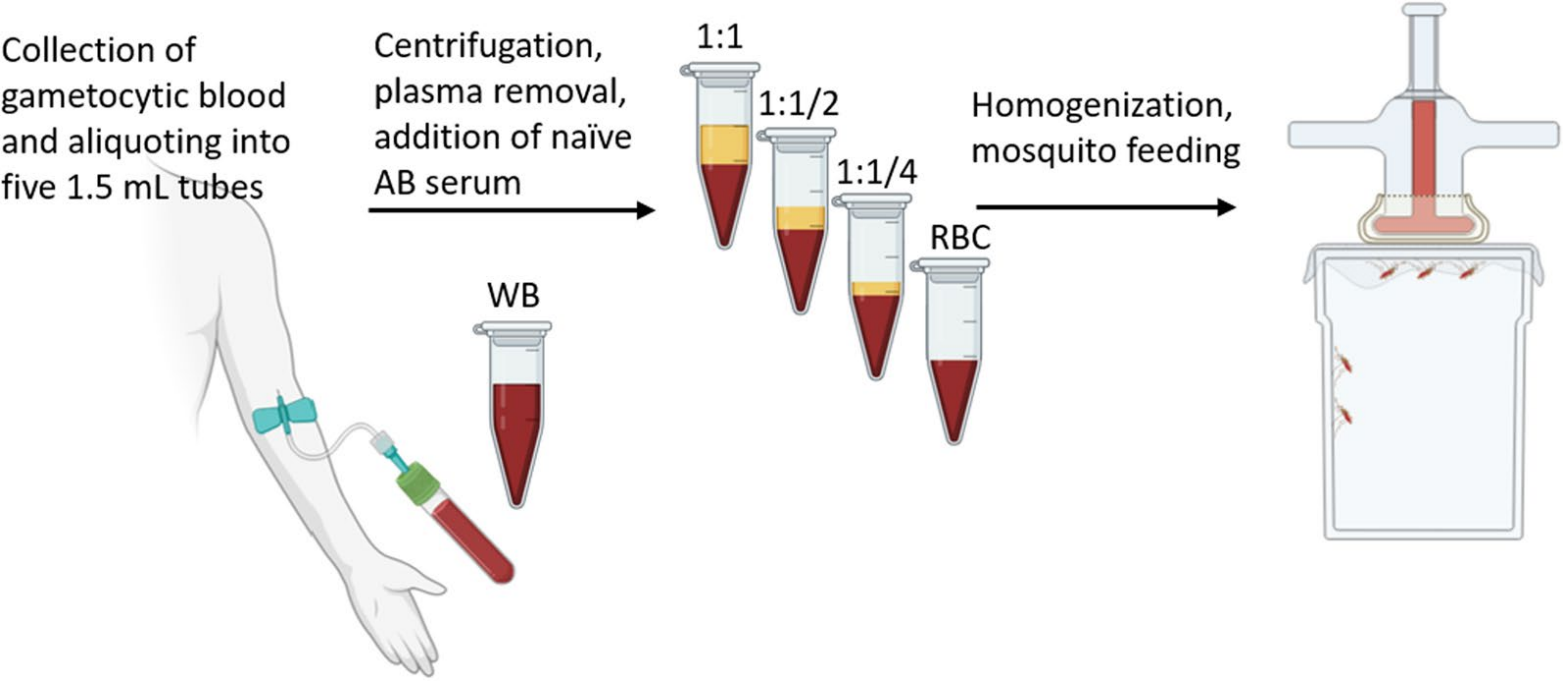
Schematic representation of the experimental set-up. Five mL of blood was collected from a gametocyte carrier into a heparinized vacutainer tube and distributed into five 1.5 mL Eppendorf tubes (1 mL/tube). An aliquot of 1 mL of the whole blood was kept for the WB treatment. The remaining four aliquots blood were centrifuged at 3000 rpm for 5 min, and the donor's plasma was removed and replaced with (i) an equal volume of malaria-naïve AB + serum (1:1); (ii) a volume of malaria-naïve AB + serum equivalent to half the initial plasma volume (1:1/2); or (iii) a volume of malaria-naïve AB + serum equivalent to the quarter of the initial plasma volume (1:1/4). The RBC treatment consisted in the sole red blood cell fraction, without the addition of serum. Following the homogenization of the red blood cell fraction and the added malaria-naïve AB + serum, five hundred μL of each treatment was introduced into microfeeders, and mosquito cups (with 80–90 individuals per cup) were positioned underneath for 1 h to allow the mosquitoes to feed. This experiment was repeated four times, each with a different gametocyte carrier

A total of 5 mL of blood was collected from each gametocyte carrier into a heparinized vacutainer tube and divided into five 1.5 mL Eppendorf tubes, each receiving 1 mL (Fig. 1). The first aliquot was kept at 37 °C for the whole blood treatment (denoted as “WB” hereafter). The remaining four aliquots underwent centrifugation at 3000 rpm for 5 min using an Eppendorf Minispin centrifuge.

For the second treatment group, donor plasma was replaced with an equivalent volume of AB + serum from malaria-naïve European donors. After centrifugation, a horizontal line marking the upper limit of the plasma phase was drawn on the tube. The plasma was aspirated with a pipette, and AB + serum was introduced up to the marked line. This control treatment causes no change in gametocyte concentration and is referred as "1:1", *i.e.* one volume of red blood cell (RBC) pellet for one volume of AB + serum (Fig. 1).

In the third treatment group, donor plasma was substituted with a volume of *Plasmodium*-naive AB + serum equivalent to half the initial plasma volume. A line at the midpoint of the plasma phase was marked, and the plasma was replaced with AB + serum up to the midpoint line. Plasma and red blood cell (RBC) volumes were nearly identical within the total blood volume, displaying minimal variation across samples. Following centrifugation, the demarcation between the RBC pellet and the plasma phase aligned with the line on the tube denoting 0.5 mL, marking the termination of the conical section of the tube. The treatment groups were named based on the fraction of initial plasma volume used to reconstitute the RBC pellet. Consequently, this third treatment group is labeled "1:1/2", meaning that the reconstituted blood sample contains 2/3 of RBC and 1/3 of serum (Fig. 1). Considering that the plasma and RBC volumes were equivalent (*i.e*. RBC:Plasma (v:v) = 1:1), this configuration results in a concentration factor of 1.33 for gametocytes (*i.e.* a 33% increase in gametocytaemia). This theoretical gametocyte concentration factor was not corroborated with observed values, as microscopic quantification of gametocytes was not conducted on blood smears from each treatment.

For the fourth treatment group, donor plasma was replaced with a volume of AB + serum equivalent to a quarter of the initial plasma volume. A line at the quarter point was marked, and after complete plasma aspiration, naïve AB + serum was added up to the marked quarter line. This treatment, resulting in a 1.6 concentration factor for gametocytes (a 60% increase in gametocytaemia), is referred as "1:1/4" *i.e.* 4/5 of the experimental blood volume is composed of the RBC pellet.

Finally, the fifth treatment group comprised only the red blood cell fraction without added serum. This treatment, denoted “RBC”, yielded a gametocyte concentration factor of 2 (a 100% increase in gametocytaemia).

A volume of 500 µL of each treatment group was dispensed into warmed glass microfeeders (Fig. 1). Paper cups containing 80–90 starved female mosquitoes were placed under parafilm membrane and mosquitoes were allowed to feed for 1 h. Non-fed females were counted and discarded, while the remaining fully engorged mosquitoes were kept in a biosafety room under the same standard conditions and were fed daily with a 10% glucose solution for seven days. Mosquito survival was recorded daily at 8 am until dissection. On the seventh day following blood meal, mosquito midguts were dissected under a stereomicroscope (LEICA® S9E, Germany), stained with 1% mercurochrome and observed under a microscope (LEICA DM1000 LED, Germany) at a × 400 magnitude to assess the presence and number of oocysts.

### Statistical analysis

All statistical analyses were performed with R, version 4.2.1 [22]. The effect of blood treatment (5 levels: WB, Ctrl, 2FI, 3FI and MI) on mosquito feeding rate (the proportion of fully-fed females) and infection rate (the proportion of females with at least one oocyst) was examined using two binomial generalized linear mixed models (GLMM, glmmTMB package [23]). Infection intensity (the number of oocysts in infected midguts) was analysed using a zero-truncated negative binomial GLMM (glmmTMB package [23]). The effect of blood treatment on mosquito survival was explored using a mixed effect Cox’s proportional hazard regression models (coxme package [24]). In these four mixed models, blood treatment was set as a fixed effect and parasite isolate (A to D) as a random effect. The ‘Anova’ function from the ‘car’ package version 3.1–1 [25] was used to estimate the significance of terms. When blood treatment was significant, multiple pairwise post hoc tests were performed to compare treatment using the ‘emmeans’ library [26].

## Results

### Feeding rate and survival

**Fig. 2:**
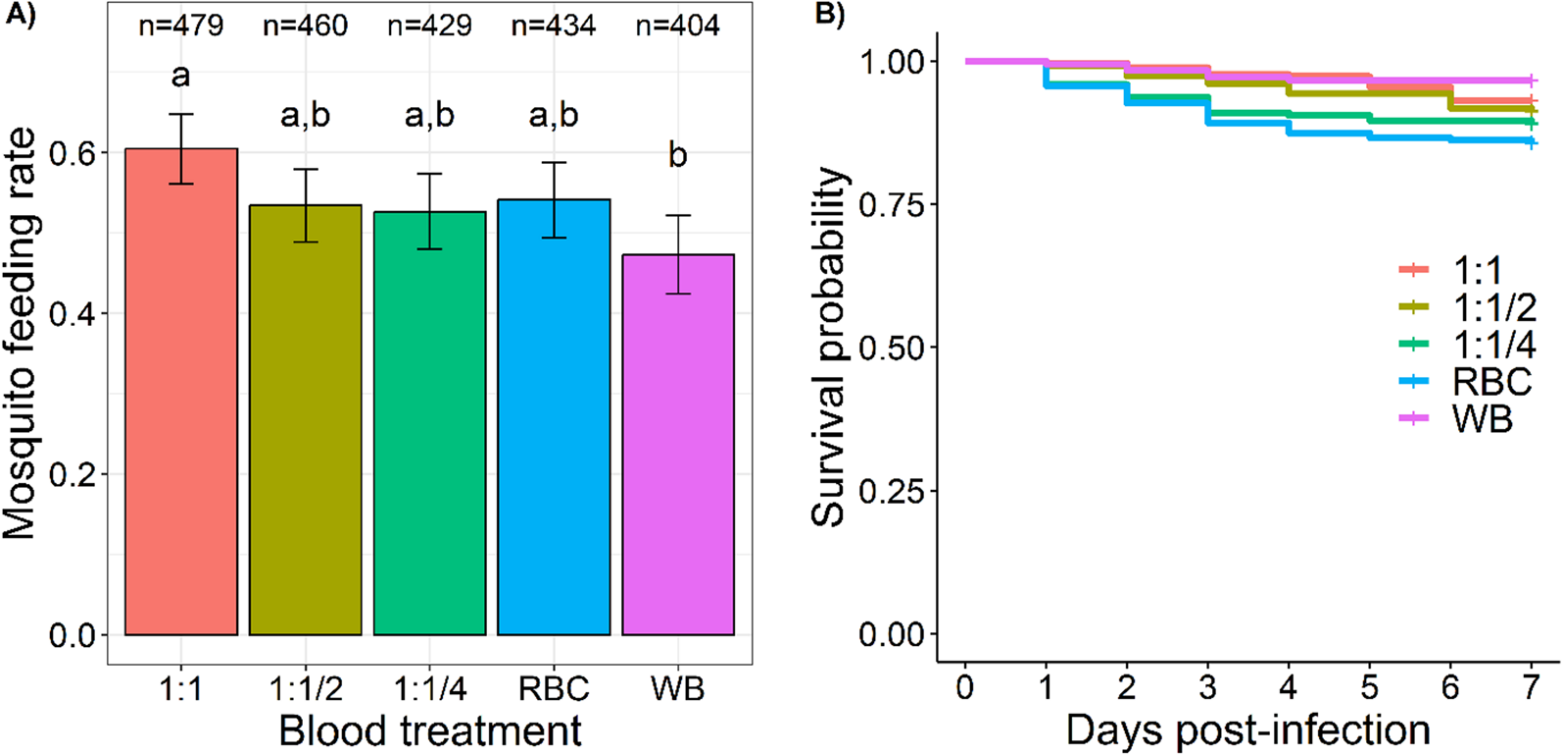
Effect of blood treatment on mosquito feeding rate and survival. A Mosquito feeding rate (± 95% CI), expressed as the number of fully blood-fed females out of the total number of mosquitoes exposed to the blood for each treatment and over 4 replicates. “n = ” indicates the total number of mosquitoes exposed to the blood. Different letters above the bars denote statistically significant differences based on post hoc multiple comparison tests. B Kaplan–Meier curves representing survival in days post blood meal for each treatment. Survival was recorded once a day from 1 to 7 days-post blood meal. 1:1: plasma replaced by the same volume of malaria-naïve AB + serum; 1:1/2: plasma replaced with a volume of malaria-naïve AB + serum equivalent to half the initial plasma volume; 1:1/4: plasma replaced with a volume of malaria-naïve AB + serum equivalent to the quarter of the initial plasma volume; RBC: plasma removed and no added serum; WB: mosquitoes that fed on whole blood

Blood treatment significantly affected mosquito feeding rate (LRT) *X*^2^_4_ = 13.6, P = 0.0087, Fig. 2A), with highest overall feeding on 1:1 control blood (60.5 ± 4%) followed by RBC (54.1 ± 5%), 1:1/2 (53.5 ± 4.5%), 1:1/4 (52.7 ± 4.8%) and WB (47.3 ± 5%). Based on post-hoc multiple pairwise comparisons, the only significant difference was between the 1:1 ctrl and WB treatment (t = 3.64, P = 0.019, Fig. 2A). Detailed feeding rates for each of the four parasite isolates (A to D) are given in Additional file 1: Fig. S1.

The survival of *An. gambiae* from 1 to 7 days post-blood meal (dpbm) was affected by the treatment (LRT *X*^2^_4_ = 18.35, P = 0.001, Fig, 2B)ith highest survival observed on WB (6 dead mosquitoes out of 178 on 7 dpbm), followed by the 1:1 ctrl (18/261), 1:2 (20/229), 1:1/4 (24/221) and RBC (33/231). The only significant pairwise differences were between WB and 1:1/4 (z = 2.9, P = 0.03) and between WB and RBC (z = 3.7, P = 0.002, Fig. 2B). Detailed survival rate for each of the four parasite isolates (A to D) are given in Additional file 1: Fig. S2.

### Infectivity

Parasite infectivity to mosquitoes was characterized by two parameters: the probability of the parasite to establish an infection within the mosquito (*i.e*., the infection rate), and the number of oocysts developing on the midgut of infected mosquitoes (*i.e*., the infection intensity).

**Fig. 3:**
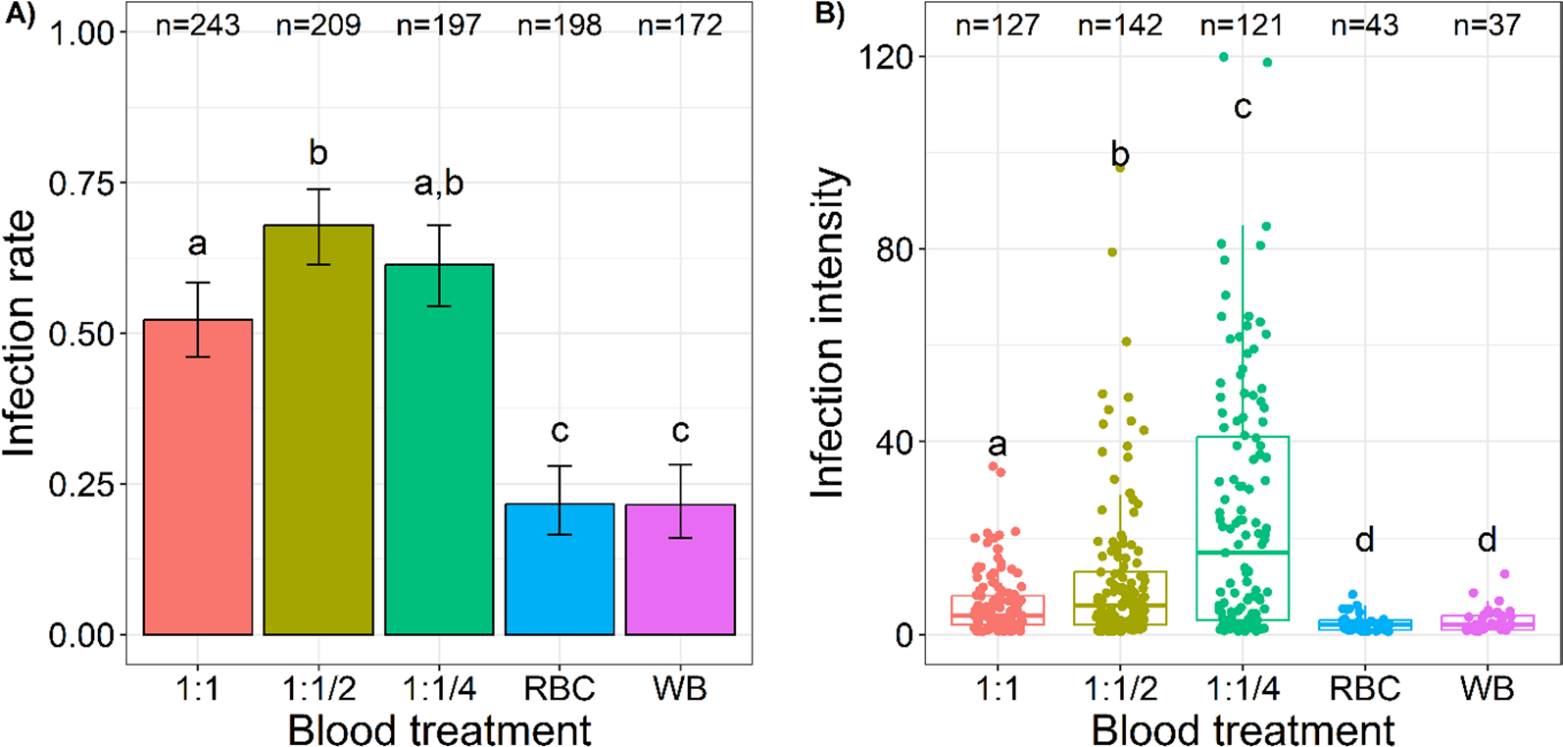
Effect of blood treatment on parasite infectivity to mosquitoes. A Infection rate (± 95% CI), expressed as the number of females harbouring at least one oocyst in the midgut at 7 days post-blood meal out of the total number of dissected females for each treatment and over 4 replicates. B Infection intensity, expressed as the number of developing oocysts in the midgut of infected females at 7 days post-blood meal for each treatment and over 4 replicates. Each replicate corresponds to a different gametocyte carrier. “n = ” indicates the total number of dissected mosquitoes in panel A or the total number of infected mosquitoes in panel B for each treatment. Different letters above the bars denote statistically significant differences based on post hoc multiple comparison tests. 1:1: plasma replaced by the same volume of malaria-naïve AB + serum; 1:1/2: plasma replaced with a volume of malaria-naïve AB + serum equivalent to half the initial plasma volume; 1:1/4: plasma replaced with a volume of malaria-naïve AB + serum equivalent to the quarter of the initial plasma volume; RBC: plasma removed and no added serum; WB: mosquitoes that fed on whole blood

Treatment had a significant impact on the infection rate (LRT *X*^2^_4_ = 156; p < 0.0001, Fig. 3A). Using half (1:1/2) or a quarter (1:1/4) of the volume of naive AB + serum led to a 31% and 17% increase of the mosquito infection rate compared to the 1:1 control, respectively (1:1 *vs.* 1:1/2, t = 3.6, P = 0.0037; 1:1 *vs.* 1:1/4, t = 1.5, P = 0.56, Fig. 3A) ompletely removing the plasma (leaving only the erythrocyte pellet, *i.e.* RBC treatment) reduced the infection rate by 58% compared to the 1:1 control (1:1 *vs.* RBC, t = 8.28, p < 0.0001, Fig. 3A) Replacing the donor's plasma with naive AB + serum increased the likelihood of infection by 143% (WB *vs.* 1:1, t = 6, p < 0.0001, Fig. 3A).

The effect of blood treatment on infection intensity was highly significant (LRT *X*^2^_4_ = 260; p < 0.0001, Fig. 3B). The mean intensity (± se) was 6.09 (± 0.57), 11.28 (± 1.27), 24.86 (± 2.35), 2.23 (± 0.26) and 2.86 (± 0.42) oocysts/midgut for the 1:1, 1:1/2, 1:1/4, RBC and WB treatments, respectively. Concentrating the infectious blood by reducing the volume of AB serum by a factor of *ca* 1.33 (1:1/2) or 1.6 (1:1/4) increased the infection intensity by *ca* 2 and 4 compared to the 1:1 control, respectively. On the other hand, keeping the natural plasma (WB) or the complete removal of this plasma (RBC) reduced the intensity by *ca* 3 compared to the 1:1 control (Fig. 3B).

## Discussion

This study aimed to adapt DMFA to enhance the infectivity of wild isolates of *P. falciparum* within its mosquito vectors through simple and practical technical modifications. The effects of five distinct blood treatments on key parameters influencing transmission potential, namely mosquito feeding rate and survival, and infection rate and intensity, were investigated. The plasma from gametocyte carriers was replaced with reduced volumes of AB + serum from malaria-naïve donors, which allowed measuring the effect of concentrating the blood mixture on parasite infectivity to mosquitoes. Such concentrations successfully enhanced the infectivity of wild *P. falciparum* isolates, without reducing the feeding rate and survival of *An. gambiae* vectors compared to the 1:1 control group. This methodological adaptation stands as a promising avenue for bolstering the transmission efficiency of the malaria parasite within its mosquito vectors, presenting new horizons for experiments on the biology of *Plasmodium-Anopheles* interactions or the laboratory evaluation of the efficacy of transmission-blocking interventions in areas grappling with low transmission scenarios.

Concentrating infectious blood with the 1:1/2 treatment (*i.e*., replacement of plasma with a volume of *Plasmodium*-naive AB + serum equivalent to half the initial plasma volume) resulted in increased of both infection rates and intensity. Relative to the 1:1 control, this 1:1/2 treatment yielded a proportional increase in the infection rate, with a 33% increase in gametocyte density (considering the plasma volume as approximately half of the total blood volume), resulting in a 31% increase in infection rate. There is a non-linear relationship between the number of gametocytes present in the blood and the resulting mosquito infection rate [13–15]. Churcher et al*.* [14] demonstrated that the infection rate's response to rising gametocyte density followed a Gompertz function; and that elevating the density from 1 to 200 gametocytes per microlitre first yielded a very marginal increases in infection rate. The prevalence data are in line with these observations, as the four isolates, once concentrated by a factor 1.33 fell within this density range (*i.e.* initial gametocytaemia of 32, 40, 72, and 80 gametocytes/μl resulting in theoretical gametocytaemia of 42.56, 53.2, 95.76, and 106.4). Beyond 200 gametocytes per microlitre, the infection rate experiences a rapid escalation [14], suggesting that the implementation of a 1.33 concentration (1:1/2 treatment) on higher initial gametocytaemia could potentially yield even greater increases in infection rates. The findings of this study also align with a prior DMFA study that investigated the impact of haematological factors of 162 natural carriers of *P. falciparum* gametocytes in Gambia. This study revealed a positive association between packed cell volume (PCV) and gametocyte infectivity to *Anopheles* mosquitoes [27].

While the rise in infection rate in the 1:1/2 treatment confirms the validity of the approach, the absence of a similar increase in the 1:1/4 and RBC treatment contradicts the expectations. The 60% theoretical increase in gametocyte density obtained by replacing plasma with a volume of AB + serum equivalent to the quarter of the initial plasma volume (1:1/4) resulted in a 17% increase only. Considering that the positive effect of the 1:1/4 treatment on infection rate was less pronounced than that of the 1:1/2 treatment, an interesting avenue for further investigation would be to examine the impact of an intermediate concentration, such as a 3:1 ratio.

The outcomes related to intensity for the 1:1/2 and 1:1/4 treatments were somewhat different. Firstly, when compared to the control, the 1:1/4 treatment yielded a notable upsurge in the number of oocysts, surpassing the impact of the 1:1/2 treatment. Secondly, the intensity analysis revealed a superlinear rise for both the 1:1/2 treatment (i.e., a 33% increase in gametocyte density led to an 85% escalation in oocyst intensity), and the 1:1/4 treatment (i.e., a 60% increase in gametocyte density resulted in a 308% increase in oocyst intensity). This differs with the linear relationship between gametocyte densities and infection intensity observed in experimental dilution assays [15]. it is noteworthy that, in these experimental dilution experiments, the proportions of RBC/plasma remained constant, with heat-inactivated gametocytic blood being used for diluting the infectious blood [15].

The complete removal of plasma, leaving only the erythrocyte pellet (RBC treatment), resulted in a 57.7% and 64% reduction in infection rate and intensity, respectively. This suggests that specific components within plasma/serum might play a pivotal role in supporting parasite establishment and development within the mosquito host. An alternative explanation is that substituting plasma with smaller volumes of serum might have elevated blood viscosity, potentially hindering optimal parasite fertilization or ookinete motility. Although not mutually exclusive, the first hypothesis appears more likely, considering that in *Anopheles* mosquitoes, red blood cells from a meal naturally concentrate through the expulsion of serum during feeding, a process known as prediuresis [28]. Consequently, *Plasmodium* parasites vectored by *Anopheles* mosquitoes have evolved in an environment with a naturally high concentration of red blood cells. To assess the relative importance of these two possible processes on infectivity, the viscosity of the concentrated treatments could be manipulated through the addition of PBS. Specifically, if substituting autologous plasma with an equal volume of PBS yields infection rate and intensity comparable to the 1:1 control treatment, it would suggest a pivotal role of blood viscosity. If not, it would imply that the parasite requires essential elements in plasma/serum for its establishment and development within the mosquito.

Elevated blood viscosity in the RBC treatment could also limit the mosquito ability to fully engorge. This could lead to smaller blood meal sizes and, in turn the ingestion of fewer gametocytes (even if the RBC treatment had the highest gametocyte concentration). Previous work showed that high PCV tend to be associated with smaller mosquito blood meal size [28–30], presumably due to increased blood viscosity requiring more energy to imbibe [31]. While the feeding rate on the RBC treatment was high (Fig. 2A) and no significant visual differences in blood meal size were apparent across treatments during the experiments, it remains necessary for forthcoming experiments to undertake a comprehensive quantification of mosquito blood meal sizes.

The replacement of donor plasma with the same volume of *Plasmodium*-naive AB + serum resulted in a substantial increase in both infection rates and intensity (*i.e*., differences between the WB and the 1:1 control treatment). This is likely due to the transmission-blocking natural immunity, whereby human acquired antibodies can bind to antigens on the surface of gametes (and/or post-gamete stages) and block the parasite fertilization and/or later sporogonic development [16, 33, 34].

The extent of the differences among treatments varied according to the different parasite isolates used (Additional file 1: Figs. S3 and S4). Multiple factors, including potential variations in gametocyte age/maturity, sex ratio, or genetic background across the four isolates, could have contributed to influencing how gametocyte concentration affected the rates of infection and intensity. For instance, future work could delve into the interplay between multiplicity of infection and the gametocyte concentration procedure to elucidate their combined impact on augmenting infectivity.

Mosquitoes fed with whole blood (WB) exhibited longer survival than those exposed to 1:1/4 and RBC treatments involving modified serum concentrations. Here again, this could be explained by either smaller meal sizes, or a complex interplay between blood composition and mosquito physiology. In particular, the absence of plasma/serum in the RBC treatment could deprive mosquitoes of crucial nutrients, impacting their physiological state and ultimately reducing survival rates.

Mosquitoes fed on the 1:1 control treatment (plasma replaced by the same volume of naive AB + serum) exhibited consistently higher feeding rates compared to those feeding on whole blood (WB) (Fig. 2A and Additional file 1: Fig. S1). This unexpected finding raises questions about the factors influencing mosquito feeding behaviour and warrants further exploration. Unique chemical cues in the plasma of the WB treatment might be less appealing, leading to decreased feeding rates. Understanding the mechanisms driving these differences could shed light on mosquito responses to varying blood compositions. This result may also be attributed to a potential technical bias: unlike the 1:1 control, the WB treatment did not undergo centrifugation. Repeating this experiment with a treatment consisting in the resuspension of the red blood pellet in its original autologous plasma after centrifugation (*i.e.* “own” treatment), could provide insights into this unexpected result.

Although the scope of this study was limited to four *P. falciparum* wild isolates and a single mosquito species derived from a newly established colony in Burkina Faso, it holds significance to extend these findings to encompass diverse *P. falciparum* endemic regions, various *Plasmodium* species, including *Plasmodium vivax*, and a wide range of vector species and populations.

## Conclusions

The primary goal of this study was to tailor the DMFA technique to suit specific experimental conditions and objectives. In this regard, the findings bear significant implications for supporting the assessment of transmission-blocking interventions in settings characterized by low transmission rates. Concentrating infectious blood using modified serum concentrations emerges as a promising strategy to amplify mosquito infection, especially in regions with low gametocyte density. This approach aligns with the demands of transmission-blocking interventions.

### Supplementary Information

**Additional file 1.** Additional figures.

## Data Availability

The data and related documentations that support the findings of this study are openly available in DataSuds repository (IRD, France) at 10.23708/F7S6I2. Data reuse is granted under CC-BY license, R script is granted under GPLV3 license.

## Abbreviations

DFA: Direct feeding assays
DMFA: Direct membrane feeding assays
dpbm: Day post-bloodmeal
GLMM: Generalized linear mixed models
IRB: Institutional review board
LRT: Likelihood ratio test
MACS: Magnetic cell sorting
PCV: Packed cell volume
RBC: Red blood cells
SMFA: Standard membrane feeding assays
WB: Whole blood
