# Supplementary material for: A simple, field-applicable method to increase the infectivity of wild isolates of Plasmodium falciparum to mosquito vectors
Source: Malar J. 2024 May 6;23:135. doi: 10.1186/s12936-024-04969-0 (PMC11075210; doi:10.1186/s12936-024-04969-0)
Supplement: Supplementary file 1 — Additional file 1. Additional figures. [file 12936_2024_4969_MOESM1_ESM.docx]

# **Additional materials**


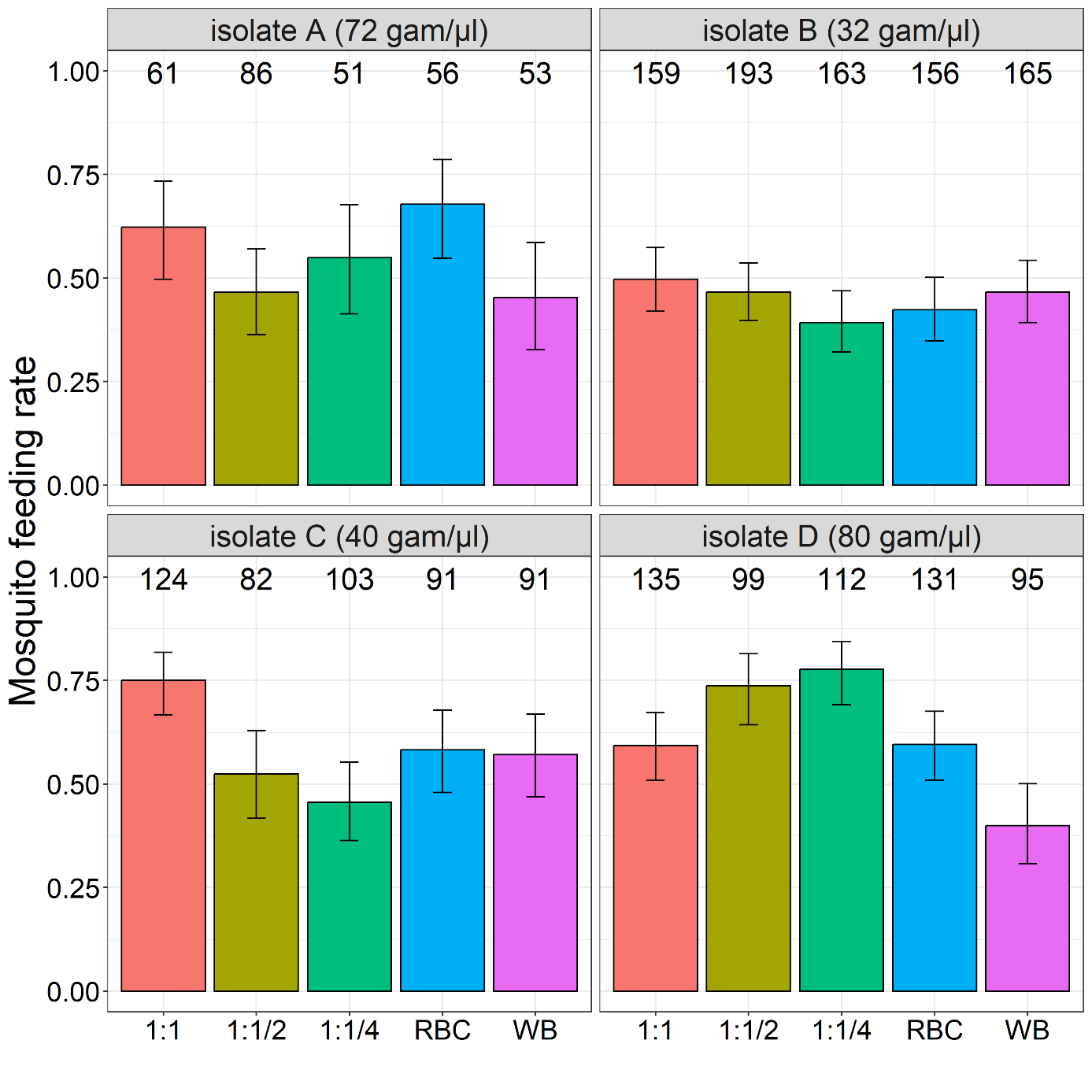


**Figure S1: Effect of blood treatment on mosquito feeding rate (± 95% CI) for each parasite isolate**. Mosquito feeding rate is expressed as the number of fully blood-fed females out of the total number of mosquitoes exposed to the blood for each treatment and over 4 replicates (isolates A to D). “n=” indicate the total number of mosquitoes exposed to the blood. WB: mosquitoes that fed on whole blood; 1:1: plasma replaced by the same volume of malaria-naïve AB+ serum; 1:1/2: plasma replaced with a volume of malaria-naïve AB+ serum equivalent to half the initial plasma volume; 1:1/4: plasma replaced with a volume of malaria-naïve AB+ serum equivalent to the quarter of the initial plasma volume; RBC: plasma removed and no added serum.

**
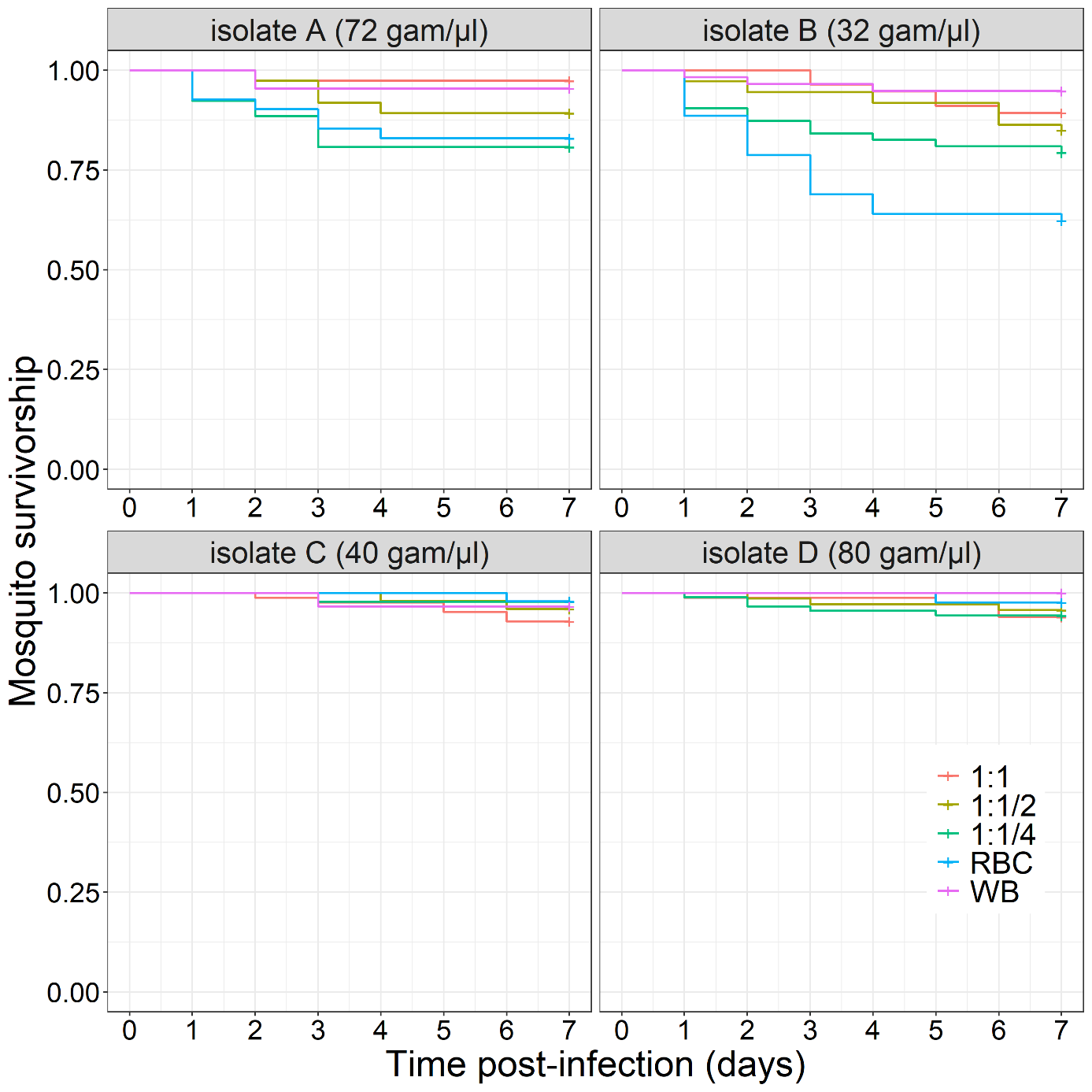
Figure S2: Effect of blood treatment on mosquito survival rate for each parasite isolate.** Kaplan–Meier curves representing mosquito survival in days post bloodmeal for each treatment. Survival was recorded once a day from 1 to 7 days-post bloodmeal. WB: mosquitoes that fed on whole blood; 1:1: plasma replaced by the same volume of malaria-naïve AB+ serum; 1:1/2: plasma replaced with a volume of malaria-naïve AB+ serum equivalent to half the initial plasma volume; 1:1/4: plasma replaced with a volume of malaria-naïve AB+ serum equivalent to the quarter of the initial plasma volume; RBC: plasma removed and no added serum.


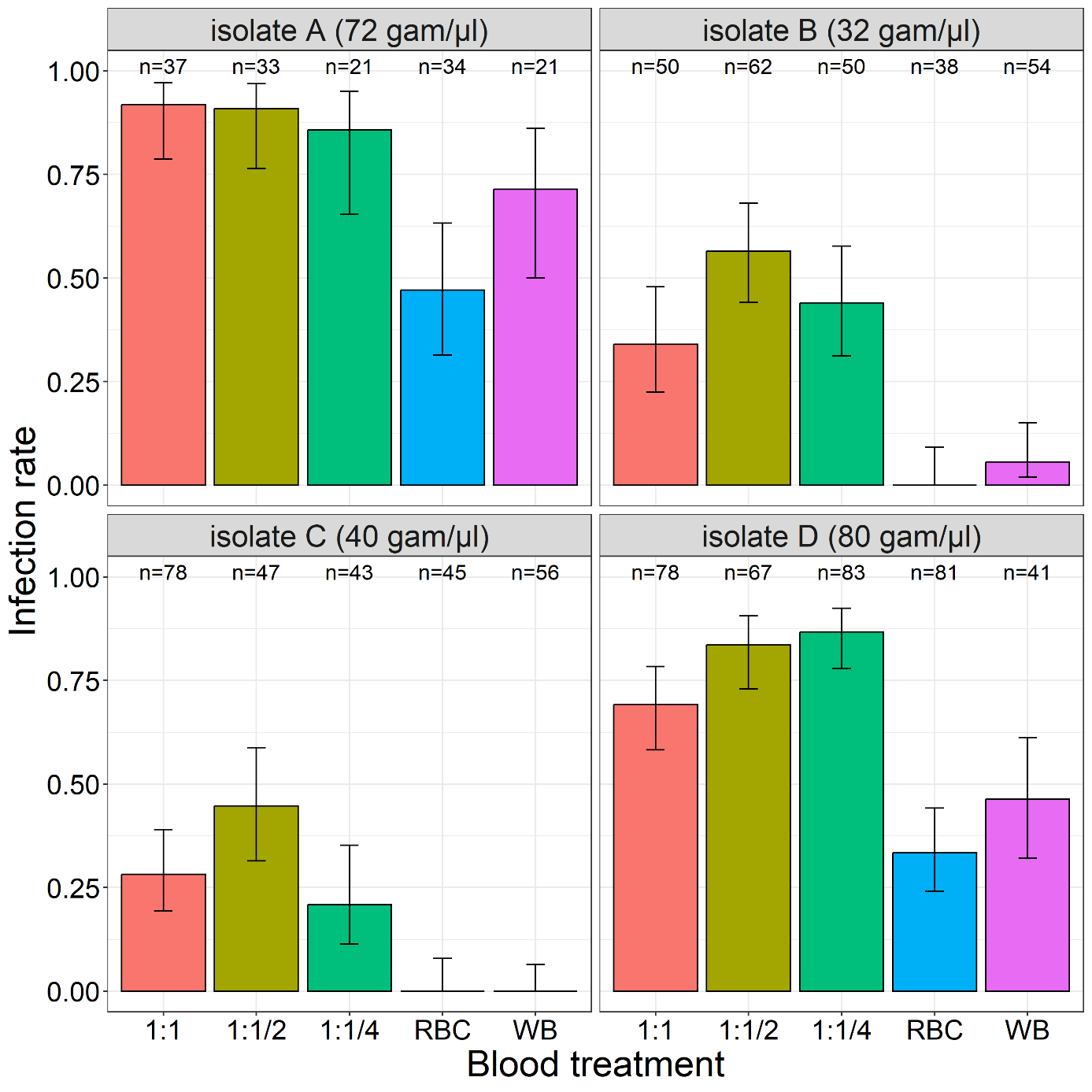


**Figure S3: Effect of blood treatment on infection rate (± 95% CI) for each parasite isolate.** Infection rate (± 95% CI), expressed as the number of females harbouring at least one oocyst in the midgut at 7 days post-bloodmeal out of the total number of dissected females for each treatment and over 4 replicates. Each replicate corresponds to a different parasite isolate. “n=” indicates the total number of mosquitoes dissected for each treatment and replicate. WB: mosquitoes that fed on whole blood; 1:1: plasma replaced by the same volume of malaria-naïve AB+ serum; 1:1/2: plasma replaced with a volume of malaria-naïve AB+ serum equivalent to half the initial plasma volume; 1:1/4: plasma replaced with a volume of malaria-naïve AB+ serum equivalent to the quarter of the initial plasma volume; RBC: plasma removed and no added serum.


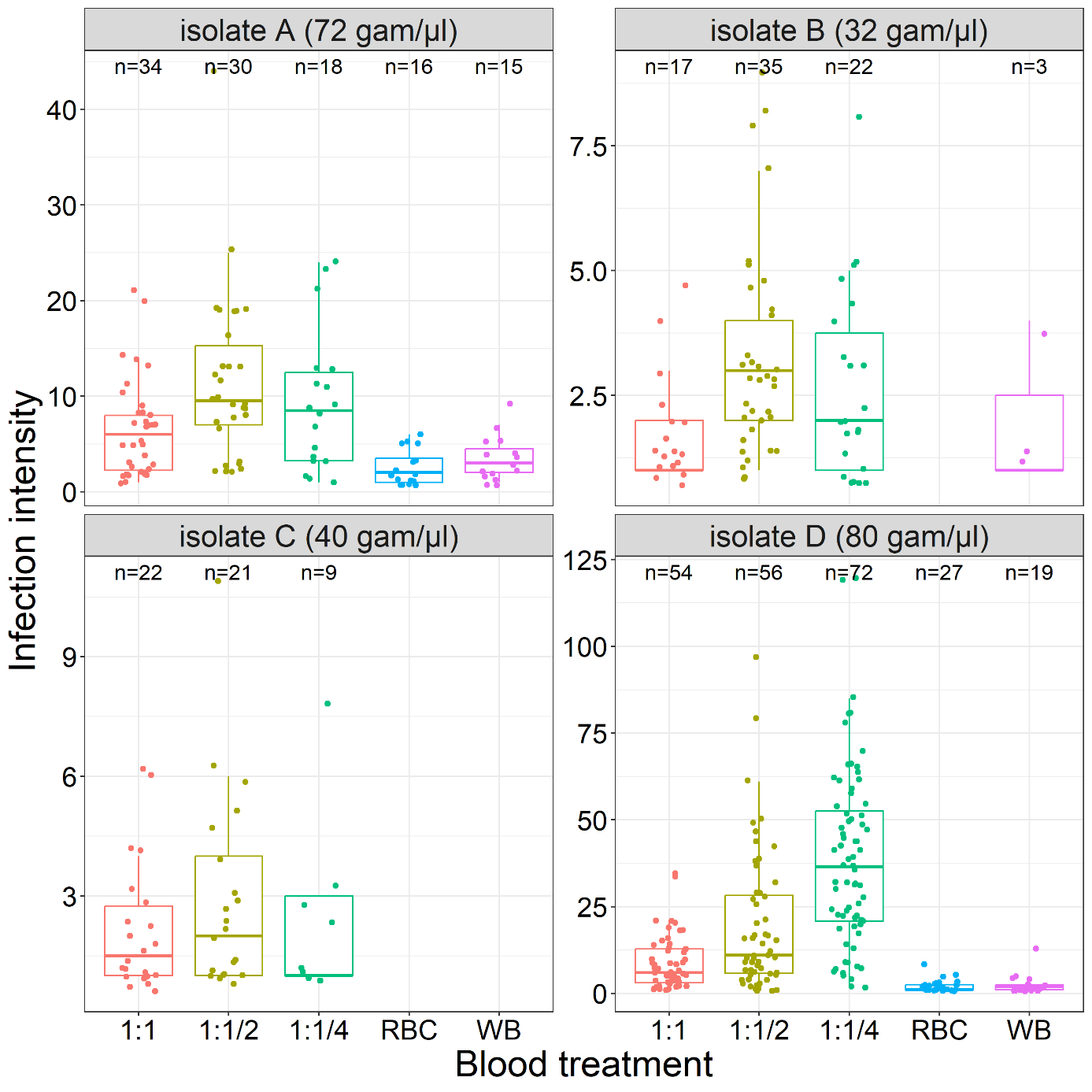


**Figure S4: Effect of blood type on infection intensity for each parasite isolate**. Infection intensity, expressed as the number of developing oocysts in the midgut of infected females at 7 days post-bloodmeal for each treatment and over 4 replicates. Each replicate corresponds to a different isolate. “n=” indicates the total number of infected mosquitoes for each treatment and isolate. WB: mosquitoes that fed on whole blood; 1:1: plasma replaced by the same volume of malaria-naïve AB+ serum; 1:1/2: plasma replaced with a volume of malaria-naïve AB+ serum equivalent to half the initial plasma volume; 1:1/4: plasma replaced with a volume of malaria-naïve AB+ serum equivalent to the quarter of the initial plasma volume; RBC: plasma removed and no added serum.
